# The Ethics of Stem Cell-Based Embryo-Like Structures: A Focus Group Study on the Perspectives of Dutch Professionals and Lay Citizens

**DOI:** 10.1007/s11673-023-10325-9

**Published:** 2024-03-13

**Authors:** A. M. Pereira Daoud, W. J. Dondorp, A. L. Bredenoord, G. M. W. R. de Wert

**Affiliations:** 1 Department of Health, Ethics and Society (HES), the Research School for Oncology and Developmental Biology (GROW), Department of Medical Humanities, Maastricht University, and University Medical Centre Utrecht, P. Debyeplein 1, Maastricht, Limburg 6229 HA The Netherlands; 2 Erasmus University Rotterdam, School of Philosophy, Burgemeester Oudlaan 50, Rotterdam, South Holland 3062 PA The Netherlands

**Keywords:** Focus group study, Lay perspectives, Professional perspectives, Ethics, Policy, Human embryo-like structures

## Abstract

In order to study early human development while avoiding the burdens associated with human embryo research, scientists are redirecting their efforts towards so-called human embryo-like structures (hELS). hELS are created from clusters of human pluripotent stem cells and seem capable of mimicking early human development with increasing accuracy. Notwithstanding, hELS research finds itself at the intersection of historically controversial fields, and the expectation that it might be received as similarly sensitive is prompting proactive law reform in many jurisdictions, including the Netherlands. However, studies on the public perception of hELS research remain scarce. To help guide policymakers and fill this gap in the literature, we conducted an explorative qualitative study aimed at mapping the range of perspectives in the Netherlands on the creation and research use of hELS. This article reports on a subset of our findings, namely those pertaining to (the degrees of and requirements for) confidence in research with hELS and its regulation. Despite commonly found disparities in confidence on emerging biotechnologies, we also found wide consensus regarding the requirements for having (more) confidence in hELS research. We conclude by reflecting on how these findings could be relevant to researchers and (Dutch) policymakers when interpreted within the context of their limitations.

**Supplementary Information:**

The online version contains supplementary material available at 10.1007/s11673-023-10325-9.

## Introduction

Research with human embryos remains invaluable to the scientific understanding of normal and abnormal human development, but it also remains an ethically sensitive practice (Svoboda 2021; Straiton 2022). The Netherlands, known for its ability to compromise in order to bridge differences (“the polder model” (Hendriks 2017)), has historically attempted to strike a balance between the burdens and benefits of human embryo research by only allowing it under strict material and procedural conditions. These conditions are stipulated in the Dutch Embryos Act (2002) and include a ban on the special creation of human embryos for research purposes as well as on their laboratory culture beyond fourteen days post-fertilization (internationally known as the 14-day rule), which effectively opens a maximum of a nine-day window (between ~E5 and ~E14) to conduct research. While these conditions limit many avenues of research, the Act has managed to enable important scientific research to continue while safeguarding the population’s confidence in this particular field of science for over twenty years. Now, twenty years later, the Act is undergoing its first significant revision (Rijksoverheid 2022).

A main reason for this revision is the recent development of—what we will refer to as—human embryo-like structures (hELS). hELS are created from clusters of human (induced or embryonic) pluripotent stem cells and seem capable of mimicking early human development with increasing accuracy and efficiency (Moris, et al. 2020; Liu, et al. 2021; Yu, et al. 2021; Zheng and Fu 2021; Chen and Shao 2022). The cellular plasticity of these structures provides unprecedented bottom-up and decoupled approaches to early human embryology (Posfai, et al. 2021), as well as the scientific ability to model stages that typically occur after multiple days of development from their very first day in culture (Hyun, et al. 2020). From a research perspective, these qualities offer a means to bypass many of the practical and legal constraints associated with human embryo research while still enabling some of its research aims. From a normative perspective, they raise the question of how to deal with the many potential loopholes brewing. In order to treat like cases alike, the boundaries associated with the use of human embryos in research should apply to hELS that have become virtually indistinguishable from them. Strict application of the 14-day rule to hELS, however, could fail to prevent the modelling of stages that lie beyond what is typically allowed in human embryo research, and extension of the ban on “research embryos” might preclude their creation altogether (Matthews and Moralí 2020). On the other hand, since these structures do not arise from fertilization nor seem (so far) capable of undergoing continuous organismal development, they currently fall outside human embryo research regulations in many jurisdictions (Matthews and Moralí 2020; Nicolas, Etoc, and Brivanlou 2021; Matthews and Moralí 2020), including the Netherlands. It is thus unclear if and how research with these structures should be regulated.

In the Netherlands, these issues have led the experts involved in the third evaluation of the Dutch Embryos Act (Dondorp, et al. 2021) to recommend (i) revising the legal definition of “embryo” to bring hELS research that attempts to model integrated embryonic development under the scope of the Act, (ii) lifting the ban on the special creation of (“research”) embryos, and (iii) reconsidering the 14-day rule. The Dutch Government plans to take up most recommendations in the current parliamentary period, but it has left the decision of lifting the ban on research embryos to a future cabinet (Kuipers 2022).

The Netherlands is not the only country that is presently revising their human embryo research legislation due to the advancement of hELS research. In the United Kingdom, the Human Fertilisation and Embryology Authority (HFEA) has recently been tasked to review existing human embryo research regulations for the purpose of law reform (Jacobson 2021), and the advancement of particular subtypes of hELS (namely, iBlastoids (Liu, et al. 2021)) has already led the National Health and Medical Research Council (NHMRC) to recommend including these structures under the scope of Australian law (NHMRC 2021). hELS research finds itself at the intersection of fields that have historically raised ethical and political controversy—stem cell research, human embryo research, and synthetic biology (Lenoir 2000; Torgersen 2009; Gouman, Vogelezang, and Verhoef 2020)—and it is therefore reasonably expected to be received as similarly sensitive, as is already the case in the United States (Subbaraman 2020), for example. The aim to anticipate these potential sensitivities by law is an important goal from the perspective of Responsible Research Innovation (RRI) frameworks (Owen, Macnaghten, and Stilgoe 2012; Burget, Bardone, and Pedaste 2017; Hyun, et al. 2021), in which the Dutch government is also heavily invested (NWO 2008). At the same time, studies on the public perception of hELS research remain significantly scarce, and the envisioned revisions may thus risk putting the cart before the horse.

This article reports on findings of a larger qualitative study that aimed to (tentatively) explore the range of lay and professional perspectives on research with hELS in the Netherlands. To the best of our knowledge, this study is the first to probe the topic empirically and can therefore help bridge the current gap in the literature by providing avenues for further research. The aim of our larger qualitative study was to probe and supplement the agenda-setting input we previously set forth and in which we mapped issues on conceptual, moral, and regulatory levels as requiring further inquiry (Pereira Daoud, et al. 2020) with the ultimate purpose of advising the Dutch government on how to proceed with regard to policymaking for research with hELS. In order to discuss relevant findings as thoroughly as possible, this article focuses specifically on themes pertaining to (the degrees of and requirements for) confidence in research with hELS and its regulation. The two remnant themes we identified in the data (on the conceptual and moral qualification of hELS) have been reported in a separate manuscript (Pereira Daoud, et al. 2022) and will be referred to in this article when necessary. In what follows, we begin by clarifying our methodological approach by expanding on the sample, setting, and analysis of the data. In the Results section, we describe the participants’ degree of confidence in hELS research, which ranged between positive, negative, and ambivalent, and the requirements they deemed necessary in order to have (greater) confidence in the field, which consisted of regulating the aims of hELS research, the development of certain features, and the involvement of the public in the advancement of the field. In the Discussion, we relate these findings to the literature, highlighting areas of common ground and mapping those in need of further investigation. We conclude on the positive note that, despite the apparent initial contention among participants, there is a large degree of consensus regarding the issues in need of further inquiry for hELS research to be societally acceptable and call fellow researchers in the humanities and social sciences to pick up these issues for further empirical research and ethical analysis.

## Methods

We performed a qualitative study with a cross-sectional design to explore the potential conceptual, moral, and regulatory issues of research with hELS. In contrast to quantitative studies, these methods allow participants to respond in their own wording, engage with each other’s views, and elaborate on the reasons supporting their own standpoints. Focus groups are particularly useful in that regard, as they have the additional advantage of making contrasts and congruencies between individual participants more perceptible and intelligible, which was important in view of the explorative research aims of our larger study. These aims were to probe and supplement the agenda-setting input we had previously set forth (Pereira Daoud, et al. 2020), in which we mapped potential conceptual, moral, and regulatory issues raised by the generation, culture, and use of hELS in a research context, with the ultimate purpose of tentatively informing Dutch policymaking. The study was approved by the Research Ethics Committee (REC) of the Faculty of Health, Medicine, and Life Sciences of Maastricht University (approval number: FHML-REC/2020/018), and a subset of its findings has been reported elsewhere (Pereira Daoud, et al. 2022). This article reports on remnant findings in accordance with the consolidated criteria for reporting qualitative studies (COREQ) (Tong, Sainsbury, and Craig 2007).

### Participant Selection and Recruitment

Given the policy and tentative aims of our larger qualitative study, we were particularly interested in collecting lay and professional (specifically, legal and ethical) perspectives. Lay participants were considered eligible for inclusion in the focus group study if they had no prior knowledge of hELS research and represented main demographic characteristics (sex, age, and educational level) of the Dutch population. The lay participants considered eligible for the pilot focus group were selected from the personal network of the first author and had not met each other previously. (APD) invited eight participants in total: first, informally via text message and, upon initial confirmation, formally via-email. Three of the selected participants ended up not joining the pilot: one for reasons unknown and two due to personal circumstances. Lay participants that were not part of the pilot were selected and approached by a professional recruitment agency and offered a small amount of financial compensation (fifty euro) for the time and effort they invested in participating in our research study. It is unknown if, and how many, participants rejected the invitation of the professional recruitment agency. Professional participants were considered eligible for inclusion in the focus group study if they had previous professional (scholarly or policy) experience in developing ethical and legal frameworks for emerging biotechnologies. Eligible participants were selected from the professional networks of the authors, and many of them were therefore professionally acquainted with one another. In total, thirteen eligible professionals were approached by (APD) via e-mail. Six of these professionals refused to participate in the focus group study: two for reasons unknown, three due to personal circumstances, and one due to a perceived lack of expertise on the topic of inquiry. Due to the COVID-19 pandemic, recruitment was kept to a minimum and ended as soon as a sufficiently diverse number of participants per focus group had been reached. All participants received the same invitational letter in advance of the interviews, which thoroughly described the topic, aims, methods, confidentiality, and informed consent procedure of the study. Written informed consent was acquired at the beginning of each interview. For an overview of the full research sample and relevant participant characteristics per group type, see table 1 and 2.

**Table 1:** Research sample of focus groups with lay participants

TYPE	SEX	AGE	EDUCATIONAL LEVEL*
		3/5 = 20 ≤ 30 years old	
FG-Lay0	2/5 male	0/5 = 30 ≤ 40 years old	2/5 ≤ MBO
(Pilot)	3/5 female	0/5 = 40 ≤ 50 years old	2/5 = HBO
(n=5)		1/5 = 50 ≤ 60 years old	1/5 ≥ WO
		1/5 ≥ 60 years old	
		3/10 = 20 ≤ 30 years old	
FG-Lay1	6/10 male	2/10 = 30 ≤ 40 years old	6/10 ≤ MBO
(n=10)	4/10 female	1/10 = 40 ≤ 50 years old	2/10 = HBO
		1/10 = 50 ≤ 60 years old	2/10 ≥ WO
		3/10 ≥ 60 years old	
		1/11 = 20 ≤ 30 years old	
FG-Lay2	5/11 male	4/11 = 30 ≤ 40 years old	5/11 ≤ MBO
(n=11)	6/11 female	2/11 = 40 ≤ 50 years old	2/11 = HBO
		0/11 = 50 ≤ 60 years old	4/11 ≥ WO
		4/11 ≥ 60 years old	
		7/26 = 20 ≤ 30 years old	
TOTAL	13/26 male	6/26 = 30 ≤ 40 years old	13/26 ≤ MBO
(n=26)	13/26 female	3/26 = 40 ≤ 50 years old	6/26 = HBO
		2/26 = 50 ≤ 60 years old	7/26 ≥ WO
		8/26 ≥ 60 years old	

*Education in the Netherlands discerns between Middelbaar Beroepsonderwijs (MBO, secondary vocational education), Hoger Beroepsonderwijs (HBO, higher professional education), and Wetenschappelijk Onderwijs (WO, higher scientific education).

**Table 2:** Research sample of focus group with professional participants

TYPE	SEX	AGE	EXPERTISE
FG-Prof	2/7 male	0/7 = 20 ≤ 30 years old	4/7 health ethics
(n=7)	5/7 female	1/7 = 30 ≤ 40 years old	3/7 health law
		3/7 = 40 ≤ 50 years old	
		1/7 = 50 ≤ 60 years old	
		2/7 ≥ 60 years old	

### Research Design and Data Collection

In order to enable the discussion of the different topics in a uniform manner whilst still enabling individual participants to raise and divagate into the issues they considered significant, we developed an interview guide (see Supplementary Files) to semi-structure the focus group interviews. The interview guide, which was developed based on the aforementioned agenda-setting input and supplemented by discussions with the research team, contained eighteen questions that aimed to probe the participants’ intuitions about hELS research in general and their perspectives on the conceptual, moral, and legal qualification of hELS in particular.

The interview guide was tested in a pilot and its first question—namely, “When you think of the possibility to create “synthetic embryos”/ “embryo-like structures,” what comes to mind? Do you think it is a positive or negative development?”—was amended later to include auxiliary imagery in order to incentivize greater discussion between participants. In order to do this in a thought-provoking yet simplified way, we used cartoon images to represent “negative” and “positive” associations. In the “negative” image, the scientist was depicted as a man with a malicious grin, wearing Doctor Frankenstein attire, while frowning his eyebrows as he forcefully held the tube in his left hand. In the “positive” image, the scientist was depicted as a woman with a friendly smile, wearing stereotypical laboratory attire, while enthusiastically pointing toward the tube she held in her right hand. This question was of direct relevance to the themes reported in this article (which we further explain below).

Due to the explorative aims of our research study and the COVID-19 restrictions in force at the time, sufficient diversity in views was prioritized over data saturation. In particular, four (in person and semi-structured) focus group interviews were conducted: three with lay participants—one of which was a pilot that consisted therefore of fewer participants (table 1)— and one with health law and health ethics professionals involved in policymaking (table 2). The interviews lasted two hours on average and were held at professional venues between the end of August and the beginning of September 2020. In each interview, only participants and the two first authors were present. (WD), a male professor and doctor with previous experience in qualitative research, moderated the interviews while (APD), a female PhD candidate with no previous experience in qualitative research, attended as an observer and practical facilitator. All interviews were conducted in Dutch, audio recorded, transcribed verbatim, and pseudonymized. The pseudonymized transcripts were not returned to participants for comments or corrections.

### Data Analysis

The transcripts were analysed thematically. Open codes were generated in Atlas.ti 8 software in an inductive or “bottom up” (rather than deductive or “top down”) manner in order to include findings that lie outside the traditional ethical discourse and that may have otherwise been dismissed prematurely (Braun and Clarke 2006). These codes were formulated as closely as possible to the participants’ own wording in order to avoid interpreting data too soon and subsequently validated by (WD) through a randomized sampling method. After that, the resulting list of open codes was clustered through mind mapping by (APD), which included interpreting individual codes in relation to the topics to which they referred as well as in the context of their respective discussions, and subsequently evaluated and adapted by the research team. This procedure went back and forth using the constant comparative method of analysis (Kolb 2012) until higher order themes could be consistently identified in the data and agreed upon by all members of the research team.

## Results

The data analysis resulted in the identification of four main themes, two of which have been reported in a separate manuscript (Pereira Daoud, et al. 2022) due to scope limitations and to which we will refer when necessary. The present article reports the two remnant themes, which we classify as issues pertaining to confidence in research with hELS and which are illustrated with quotations (in relation to group type, respondent number, theme, subtheme, category, and code, if applicable) in table 3 (see online supplementary materials).

**Table 3:** Illustrative quotations per group type theme, subtheme, category, respondent number, and, if applicable, category. *Lay0* stands for the pilot focus group with lay participants, *Lay1* and *Lay2* stand for the two remaining focus groups with lay participants and *Prof* stands for the focus group with professional participants, as per Table 1 and 2

Nr.	Group	Subtheme	Category	Translated Quote	
***Theme I: Degrees of Confidence in hELS-Research***					
1	Lay0	Positive Perspectives (about)	The Utility of Research	**R04**: “Suppose it is ... so that we do not have to test on animals, I think it's good.”	
2	Lay1			**R05**: “Yes. For me, it also goes more towards the [positive image]. ... I think it is very nice, so to speak ... that something like this can be developed. So I would rather go towards the [positive] picture, indeed.”	
3	Lay2			**R04**: “For example, I am thirty and my fertility is declining more and more after my thirtieth birthday. But society has also changed so much that I am no longer settled at the age of 22 and have a partner with whom I have a house and will stay with it for the rest of my life, for example. So that, when you put it that way, I think it is quite a tricky issue. That I think, yes, because of how our society is organized, my life has turned out in such a way that I cannot or do not want to start having children in my most fertile period, so to speak. So then I think ... a method like this, IVF, offers a very nice solution for this situation.”	
4	Prof			**R02**: “Well, […] with the foreknowledge that I have of course, I'm [leaning] a little more toward that [positive image] because I do indeed see it as an opportunity to do embryo research at an early stage of development without having to use embryos that maybe were once intended for reproduction or that could be used for that purpose.”	
5	Lay1		The Researchers	**R10**: “Well, I think [the] left picture applies ... I actually think [that] everyone professionally involved in this has good intentions [and] that no one wants to abuse the techniques.”	
6	Lay2			**R02**: "But I think most scientists, in principle, want the best for ... humanity or, you know, do their work from a positive motivation."	
7	Prof			**R05**: "So the view I have of science is not [one] where you have to be incredibly wary that [scientists] are going to do all sorts of crazy things that we don't want [them to do]."	
8	Prof		The Governance of Research	**R05**: "But of course you always ... have a monitoring system in science and, actually, my positive view of this slide had also to do with that. That ... I have quite a lot of confidence in that [system]."	
9	Lay0	Negative Perspectives (about)	The Utility of Research	**R01**: "No, I stopped that. I could have [had] it, but I did not want it myself. I found it too intense. And I think it is also a bit like ... it comes as it comes. So, it is also fate, right? ... I always say [and] I'm not religious but some things are apparently good for something."	
10				**R10**: “But if it doesn't work out, you have to accept that, no matter how difficult that is. You can also adopt children."	
11	Lay1			**R03**: "I also think there [is] a difference ... whether it [is] about ... saving human lives, instead of making human lives."	
12	Lay2			**R01**: "That documentary series, ‘Beter dan God’, of why people always have to have a nose job … or a-this correction or a-that correction. At some point, accept your body as it is."	
13	Lay1		The Researchers	**R03**: "[Suppose] that you have a complete embryo that can then grow into a human being, I find that ... scary. Because then there only needs to be one such Frankenstein and things go wrong. So we can make very good agreements about that, but [that possibility alone] is already nerve-wracking, I think."	
14				**R04**: "Three glasses of wine is good and the other researcher says you shouldn't drink wine at all. How far does the researcher’s knowledge go in this area? Yes, science is not always ... I don't know, you know."	
15	Lay2			**R01**: "And we're focusing on the Netherlands right now, whereas I think, if you look globally, there are plenty of regimens that, if they could implant certain things into human brains, would benefit from that. [I] think it's a bit silly to refer to doctor Mengele ... but that's the nightmarish sight that [hELS-research] arouses in me."	
16	Prof			**R05**: “[Jiankui] He is, of course, the example in another field, but whom has nevertheless just gone ahead and actually done something … that the entire international community condemns, right? So, it can happen. ... That is something we need to consider.”	
17	Lay1		The Governance of Research (due to)	Resignation	**R08**: “Yeah, you can’t stop technology anyway.”
18	Lay2				**R01**: “That is, of course, you cannot get a grip.”
19	Lay2			Cynicism	**R01**: “I'm suspicious anyway. What is it called? Pink Floyd has a nice song in which Roger Waters sings, "Mother, shall I trust the government?" I do not know if you have ever been to a Pink Floyd or Roger Waters concert, […] there are those giant screens with "never trust the government". And that's part of it for me.”
20	Lay2			Lagging behind	**R01**: “But ... that confirms to me the image of, yes, give science the time and resources and they will experiment away, to put it bluntly. … I want to formulate it more neutrally. But you are going to give science means without [having] criteria or legislation beforehand. So, as a legislator, you are always lagging behind the facts.”
21	Prof				**R01**: “You make ... a law, an Embryo law ... and then all of a sudden comes this, and then you’re simply lagging behind events. And how should you then deal with it? Actually, this topic [hELS-research] illustrates it already.”
22	Lay2			Shifting norms	**R07**: “I think it's very… Well, on the one hand, it can be very good to use [hELS] ... but on the other hand, a slippery slope.”
23	Prof			International arrangements	**R07**: “What I find difficult ... is that I do think that [hELS-research] is of course something that happens internationally. So I think that is really very complex. … We can do all sorts of things in our small country and adjust our Embryos Act ... but I think yes, this really presupposes some kind of arrangement on an international level and also governance on an international level.”
24	Lay2			Commercial use	**R02**: “And I think there's a handful of people who invest in [hELS-research] and who want to make power [and] money out of that and ... who don't really care much for ethics. And I think that ... that's the problem.”
25					**R06**: “Yes, especially that ‘determining’. Because in other circumstances I can imagine that [hELS-research] could have a function in commerce.”
26	Prof				**R05**: “That is exactly ... what I did not say but do realize, [namely] that if I have concerns about anything … it is the role of commercial parties and the commodification of human tissue”.
27	Lay1			Reproductive use	**R03**: "[Suppose] that you have a complete embryo that can then grow into a human being, I find that ... scary. … Well, yes, if [it] falls into the wrong hands. That would... Yeah, you see it in movies [like ‘The Boys of Brazil’] sometimes, to be able to train people … or something like that.”
28					**R09**: “I have to say that I find the idea of ​​a clone or fully-grown [hELS] odd, but not exactly scary, because I don’t really know … why it would be bad. I think it's strange, but … I don't find it scary per se.”
29	Lay2				**R01**: “I thought about cloning. If that were to be [possible], that people could be cloned with [hELS], then you really go in the direction of the [negative] image.”
30					**R07**: “Yes, there is a crux for me. I have been thinking about … what does that mean for me? If [a hELS] could actually become a human being? ... Then I'm like ... that [is] just so different, that it doesn't feel ethically right to me.”
31	Prof				**R07**: “The only real concern I would have is that cloning might be coming very close. And I have some doubts about that, [about] whether that it is a good idea.”
32	Lay1	Ambivalent Perspectives (due to)	The moral indeterminateness of research	**R02**: “Yes, it seems to me an advocate and an opponent … The female [an] advocate and that male an opponent.”	
33				**R03**: “For me there is a type of slider between [the positive and negative image], so to speak. So [it is] not [just] one or the other. ... And for me it leans towards that pretty lady on the [positive] image. But there is no rose without a thorn.”	
34	Prof			**R01**: “I actually see it as ... two sides of one and the same coin. Science brings good things, but [it] also brings things that are not good. … And a positive effect can simultaneously be a negative effect ... and vice versa. [So] maybe it is just [a matter of] ... from which perspective you look at it, whether something is right or wrong, isn't it? Because that also presupposes a kind of moral starting point.”	
35	Lay0		Comparative knowledge or lack thereof	**R01**: “Doubt. Good and bad, but that is more because you do not know what it means. 'Synthetic' makes me think ‘that's not real’. On the other hand, as I read [the information letter], those embryo-like structures are created from, say, my cells. So is it really life after all? Or is it … something that, say, has no sentience?”	
36	Lay1			**R06**: “Yes, I would lean towards the [positive] image, but that is more because it there is something naive and uninformed about it. So, for me, that image indicates that we are working on something but that we have virtually no idea what the implications are.”	
37				**R07**: “Well, maybe I can add to it. Because I think I have a bit of the same thought. I also lean towards the [positive] image. But that's more because I don't know much about this subject. And my outlook on it is [that] I am in favour of science. So I would say, do as much research as possible. But yeah, I don't know much about it. So I don't know if that's a valid reason.”	
38	Prof			**R03**: “… the most relevant issue is, I think, that we need to make a strong case that these kind of things can contribute a lot, or sufficiently, to what we are not able to do yet, right? That there are no alternatives for it that may arouse less of an image of science as a futuristic endeavour …”	
39				**R06**: “Are there possible alternatives conceivable? I also just immediately started thinking, how does this relate to organoid research, for example? Because I think you could also achieve some of the aims with organoids …”	
***Theme II: Requirements for Confidence in hELS-Research***					
40	Lay0	The Need for Regulation		**R02**: “ ... I think that those human embryo-like structures should definitely also be regulated by law. Not in the same way as human embryos, I think. Or maybe [in the same way, even though] I don't think so. Because now it does not fall under the law, so you can do anything with it. And assuming that [ELS] may become viable, that [they] may develop sentience, et cetera, it should be regulated. Because otherwise you will end up running into problems.”	
41	Lay2			**R06**: “Absolutely. In order to guarantee that [hELS-research] stays within certain limits; must stay within limits.”	
42				**R04**: “And strict regulation.”	
43				**R07**: “Yeah. And a new category.”	
44				**R09**: “Being careful with life.”	
45	Prof			**R01**: “Well, I'd rather, at least provisionally, see some kind of separate protection regime. I think the idea of it being unprotected, is indigestible. Because that means that you are actually also creating the possibility for scientists to endlessly experiment with it. And maybe eventually make a human being out it. […] So you have to contain it somehow. It could be that at a certain point we get to a situation in which we have to say [that ELS] are viable. Well, then it automatically falls under the Embryos Act. […] [But] if the consequence of the definition in the Dutch Embryos Act is that [ELS] completely fall outside of it, then I think that is an undesirable situation. Because I think there should also be some kind of protection afforded to it. Even if only in the context of surveillance by Medical Ethics Committees, [informed] consent, and such-like queries.”	
46	Lay2	Requirements for Regulation	Limit Research Aims	**R08**: “Yes. I would like to add, I think it really depends on how [hELS-research] is used.”	
47	Lay1			Commercial aims	**R10**:** “**If [hELS-research is used] for commercial purposes, then it is objectionable. But if it can help people and spare suffering ... Yes, then I think the aim is justified.”
48	Lay2				**R03**: “Look, people are curious, I think- Right, I assume science has [our] best interests at heart, in the end. But I see it differently for commerce.”
49					**R11**: “Especially if you look at the current behaviour of the pharmaceutical industry.”
50	Lay0			Eugenic aims	**R01**: “ ... because you are not sure, but also perhaps fear, the danger that they can achieve that. I find that very scary. And then I would say no, we just won’t do it. Because ... something completely different, just like Hitler, right? He also only wanted blonde and blue eyes. And he wanted to create the perfect race. Do we want to go there? That kind of stuff can happen, right? Like you were saying, you already have people saying … ‘I want a donor and I want that person to be educated and have dark hair and blue eyes.’ … I do not know. It [would be] such a perfect, no fun world.”
51	Lay1				**R01**: “That you are not going to use [hELS research] to have a boy [or] girl or brown [or] blue eyes or hair colour. Only for, yes, medical purposes that are important. Health. That you might be able to bypass hereditary diseases.”
52	Prof			Added value	**R03**: “ ... so I think that the argument … that this is really something that contributes to the societal interest, … is, pragmatically speaking, important in [avoiding] depictions of science as the mad scientist”.
53					**R05**: “No, but still, even if you were not to build [suicide genes] in, I still think preference should be for those embryo-like structures. Because you do not need oocyte donors for that. … Look, if you can simply make those embryo-like structures from a bit of material you already have, then you don't have to ask me which is preferable.”
54	Lay1		Limit Developmental Features	**R10**: “I think [regulation] is very important for [research with] embryos. For embryo-like structures, it depends on how they develop. If they remain a cluster of stem cells, then I think it is less important than if that cluster starts developing lungs by itself and continues to develop itself into a mini person. For me, that’s an important distinction.”	
55	Lay1			Heart(beat)	**R05**: “Well, if it is not the case in an embryo-like structure that … the heart starts beating, then you can do research for longer [periods of time]. But the moment that heart starts forming, I say until there and no further. … Then it is life. … If my heart stops [beating], my life is over.”
56	Lay0			Pain receptors	**R01**: “ … if a nervous system comes into being, then we get back to that sense of maybe it can feel something … Then I would say, for me, that is the limit.”
57	Lay2				**R02**: “Yes. I think ethics depends on [the ability to feel]. We do not need to talk about … bricks. Because bricks are bricks, right? But the moment a live [being] feels something, you can start to wonder how you should deal with it.”
58					**R07**: “I just said, at one month [of development] there is no brain development. [So] I would not have a problem with it.”
59	Lay0			Developmental potential	**R01**: “So, I think [that] that is really a line, also in the law … like, [hELS] should not be able to grow into a … human thing. … there must be that limit.”
60	Lay1				**R09**: “I also think—we also have that with those embryo-like structures, don’t we?—that it depends on … what you let it grow into, what it will become. That that is very important. Is it going to be an embryo or is it going to be something that looks like it but that cannot really develop into [a human being]?”
61	Prof				**R01**: “But if it cannot become a human being, then I still think that that embryo, or that structure, needs a certain amount of protection.”
62					**R04**: “Look, as long as [hELS] cannot yet grow into a human being, I think that is of a different order than when that [is possible]. For me, there really is a bit of a limit, whether you can ... develop those embryo-like structures ... to such an extent that they [...] could grow into a human being. For me, [that is where] there is a kind of breaking point.”
63					**R07**: “No, the entity is different as far as I'm concerned because I attach great importance to the moral bearing of that potential to develop [into a human being]. ... And if that’s not there at all, then I wonder: what exactly are you protecting? Then [ELS] are merely cells, and you are definitely allowed to do with it as you please.”
64	Lay1			Precaution	**R03**: “No, no, because then ... it does become a human being, [but] only to a certain extent. Until a certain age.”
65	Prof				**R07**: “Well, then you have to take that into account now and say, then we're going err on the safe side of safety and not give [hELS-research] a kind of cart blanche under the motto 'it's not an embryo'. Then you have to say ... we will set similar requirements. From which requirement numero uno is that you do not let it grow [a human being].”
66	Lay0		Involve the Public		**R01**: “We get more insight [into hELS-research] and [researchers] get more insight into how people think about it. What they find of it.”
67					**R03**: “Yes, sometimes it is good to involve the public. That way you can also get other ideas, other perspectives.”
68					**R08**: “Yes, I think it is actually important that society has an opinion about this. I mean, assuming that there will be [legislation for hELS-research], that [legislation] must of course also be a kind of reflection of what society thinks. … So I think it is important that [the public] feel[s] heard.”
69	Lay1				**R09**: “Inform people more about what [hELS-research] actually is, what it … entails.”
70	Prof				**R01**: “Because … we do not know what exactly constitutes a good development. … I think we have no choice but to societally determine what that development should be. And that is what we then call a good development.”
71					**R06**: “ ... that is a discussion … you also want to have it on a societal level. That [discussion] is not something you want to leave up to individual scientist alone.”

## Degrees of Confidence in hELS research—Positive, Negative, and Ambivalent Perspectives

In each group, participants had very different perspectives on science in general and hELS research in particular: whereas a number of participants considered the ability to create and use hELS for research purposes a positive development, arguing that they believe such endeavours will be beneficial, others were sceptical and favoured caution over enthusiasm. In between these extremes was a large group of participants with ambivalent feelings, doubting the extent to which they do or do not approve of hELS research. In what follows, we describe these results and expand on the particular reasons for the participants’ degree of confidence in the field. When appropriate, we distinguish between the motivations of lay and professional participants.

### Positive Perspectives About hELS Research

Participants with a positive outlook on hELS research expressed that they believed hELS could contribute to new insights into developmental disorders and fertility problems, while potentially alleviating a great deal of suffering—for example, by minimizing the use of animals and perhaps even altogether replacing the use of human embryos in research (table 3, quotes 1–4). These positive expectations were also explicitly associated with the participants’ confidence in both the researchers and in the ability of society to monitor and control the further development of hELS (table 3, quotes 5–7). For professionals, their positive outlook on hELS research had additionally to do with their confidence in the regulatory systems in which science is already embedded (table 3, quote 8).

### Negative Perspectives About hELS Research

Among participants with negative intuitions about hELS research, several expressed scepticism with regard to the utility of the field. For some, this had to do with preferentially allocating scientific efforts to more pressing human needs (table 3, quote 11). For others, it had to do with hELS research being perceived as yet another hubristic attitude toward human life, with some participants stressing that one should learn to accept one’s reproductive (mis)fortune, rather than continuously strive for improvement (table 3, quotes 10 and 12). This conviction that infertility should be accepted was notably shared by an involuntarily childless participant (table 3, quote 9) and led to the sharing of similar understandings in that group, the thrust of which was that scientific efforts to engineer the human condition are not necessarily for the best.

In addition to concerns about the utility of hELS, negative perspectives also arose in view of reservations about scientists. For some, these reservations had to do with a general scepticism about scientific knowledge fed by experiences with contradictory scientific claims (table 3, quote 14). For others, it had to do with the particular fear of hELS researchers feeling inclined to go beyond what is socially acceptable and deliberately pursue unscrupulous aims. The concern about “scientists going rogue” was especially perceptible in lay groups (table 3, quotes 13 and 15), but it was also acknowledged by professionals, one of which referred to Jiankui He, a Chinese scientist who became worldwide news for having prematurely used germline editing in birthed humans, as the epitome of a science cowboy (table 3, quote 16).

Another perceptible reason for hesitancy toward the field was the presumed inability of society to monitor and control the further development of hELS research. In lay groups, general utterances seemed to conceive of emerging biotechnologies as inevitable and uncontrollable forces of disruption (table 3, quotes 17–18), a finding that captures the broad sense of unease we felt during lay discussions and presumably indicates a certain sense of public resignation. While one of these participants expressed that his sense of unease had to do with his cynical outlook on politics in general (table 3, quote 19), most participants seemed more concerned about the practical feasibility of setting limits to scientific developments. This concern had to do with three main challenges: (i) regulative terms and definitions being quickly outdated as a result of new scientific developments (table 3, quotes 20–21), (ii) scientific developments continuously shifting previously set and societally agreed upon norms (table 3, quote 22), and (iii) rules for research with hELS requiring consistent application across international jurisdictions (table 3, quote 23).

The concern that it may not always be feasible to regulate hELS research in view of these challenges was particularly salient with regard to the commercial and (hypothetical) reproductive application of hELS. Participants worried that commercial uses may cause the development of hELS research to be driven by financial interests, such as those of the pharmaceutical industry, rather than by the interests of society and humanity (table 3, quote 24). Moreover, one of the participants in the professional group feared that the commercial application of hELS might lead to or otherwise encourage the commodification of (sensitive) human material (table 3, quote 26). Finally, lay and professional participants were also perceptibly wary of using hELS for reproductive applications, often because this would amount to a form of cloning, which was perceived by many as morally wrong (table 3, quotes 27, 29–31). The reasons for viewing hELS cloning as frightful or fundamentally wrong were not spelled out by these participants, nor were they always immediately clear to others in the group (table 3, quote 28). The categorical nature of these rejections during the discussions, however, lead us to hypothesize that they must relate to fundamental questions about human identity and human existence, rather than about offspring risks.

### Ambivalent Perspectives About hELS Research

Several participants also expressed having ambivalent feelings about hELS research, with most of them arguing that, despite being inclined towards a more positive outlook, they would have preferred there to be a “slider” in between the positive and negative images we presented them with (table 3, quote 33). This ambivalence arose due to (i) scientific research being perceived as morally indeterminate, and (ii) (lack of) knowledge about comparable emerging biotechnologies.

On the former, participants explained that they did not immediately have positive or negative associations, because that would depend on how hELS research is used: both lay and professional participants noted that scientific research may be used for good and bad purposes, and that research with hELS can similarly work both ways (table 3, quotes 32–34). The fact that these participants did not conceive of hELS research as an “either/or” but as an “and/and” endeavour, suggests that it is the perceived moral indeterminateness of research that lies at the heart of their mixed feelings towards this particular field.

On the latter, there were notable differences between lay and professional participants. Whereas, in lay groups, participants explicitly indicated that their doubt had to do with not knowing enough about hELS research (table 3, quotes 35–37), the professionals’ immediate contemplation of potentially preferable alternatives (table 3, quotes 38–39) suggests that their hesitation arose instead from their high familiarity with comparable debates and alternative biotechnologies.

### Requirements for Confidence in hELS Research—Regulating Aims, Features, and Public Involvement

The common ground between lay and professional participants was much greater when it came to their requirements for having (more) confidence in hELS research. Differences in expertise levels did not seem to get in the way of reaching a general sense of consensus, with both lay and professional group emphasizing the importance of developing at least some regulation for hELS research and suggesting similar regulatory limits. On the unclear legal status of hELS, for instance, most lay and professional participants agreed that insofar as hELS are incapable of further development they are indeed not human embryos in terms of the Dutch legal definition, which defines the embryo as “a cell or cluster of cells with the potential to develop into a human being” (Embryos Act 2002). Nevertheless, both emphasized that this alone should not preclude them from being due at least some degree of legal protection. How much protection they should be due depended in turn on their different views on the conceptual and moral qualification of hELS (Pereira Daoud, et al. 2022).

#### The Need for Regulation

Despite lay and professional groups agreeing that there should be at least some regulation for hELS research (table 3, quotes 40–46), the technical know-how on how to develop said regulation was understandably more pronounced among professionals. Whereas lay participants generally relied on broad recommendations (table 3, quotes 41–44), professionals specifically urged policymakers to adapt the Dutch Embryos Act in ways that allow drawing a distinction between research with embryos and hELS, while still affording (different degrees of) protection to both (table 3, quote 45). There was discussion in lay and professional groups about which features this distinction should be based upon (Pereira Daoud, et al. 2022), but both groups argued nonetheless in favour of similar regulatory boundaries. In what follows, we discuss the three regulatory conditions our lay and professional participants proposed in order to safeguard their confidence in hELS research, namely that proper regulation should (i) limit the aims of hELS research, (ii) restrict the development of certain features in hELS, and (iii) enforce that hELS research engages and develops in line with public norms and values.

#### Regulation Should Limit the Aims of hELS Research

The domain of application of hELS research was an important consideration for both lay and professional participants, with people arguing that their confidence in the field would largely depend on how hELS research is used (table 3, quote 46). Participants were adamant about regulating the purposes of hELS and limiting them to important or worthwhile ones only. Commercial (table 3, quotes 47–49) and eugenic (table 3, quotes 50–51) purposes were often discussed in striking contrast to what was perceived as worthwhile.

The general concern evoked by commercial purposes seemed again to be that the financial interests of commercial parties would ultimately trump the beneficial uses that hELS research could have had for the health and well-being of people in general (table 3, quotes 55–57). Other participants were more nuanced, with one arguing that there may be a useful role for commercial investors as long as their interests are not allowed to determine the aims of hELS research (table 3, quote 25). Taken together, however, the use of commercialization as an example of potentially undesirable applications of the technology suggests that hELS research may lose societal support if its aims are perceived as being (exclusively) profit-oriented rather than people-oriented. From a policy perspective, it could be inferred that constraining the degree to which hELS can be used for financial gain may provide a way to appease this concern.

Eugenic purposes were similarly perceived as being incompatible with what counts as worthwhile, like gaining insight into hereditary diseases (table 3, quote 51). The importance of regulation curtailing eugenic aims was not further specified, but it seemed again to connect with the (so far, hypothetical) idea of potentially using hELS reproductively. The example of future parents choosing between rather trivial physical characteristics in their offspring is a familiar trope, and it may suggest that eugenic aims were seen as a matter of catering to mere reproductive wants, rather than serving actual human needs.

Finally, on the condition that research aims must be of “added value” for society in order to be worthwhile, everyone seemed to agree, but there was some debate among professionals about whether hELS research could be of added value in jurisdictions that allow the creation of research embryos (table 3, quotes 52–53).

#### Regulation Should Limit Developmental Features in hELS

The participants’ approval of research with hELS also depended on the degree in which these structures mimic the presumably morally relevant features of human embryos (Pereira Daoud, et al. 2022). Participants were particularly concerned with exhibits of organogenesis and developmental potential, arguing that both should be designated by law as cut-off points for hELS research. Cut-off points in organogenesis were predominantly linked to features associated with the ability to feel pain and emerging consciousness, such as the development of the nervous system and (early) brain (table 3, quotes 56–58), but the development of the heart was also mentioned in one of the lay groups (table 3, quote 55).

Developmental potential, or the ability to successively progress through distinct human stages, was also given considerable thought in every group (table 3, quotes 59–63), with both lay and professional participants agreeing that regulation should restrict the creation of hELS with developmental potential (in the sense of being “viable” or able to grow into a human being). Nonetheless, their reasons seemed to differ. Professionals were noticeably more outspoken about the degree of moral reverence this feature would and should involve. Whereas some professionals argued that non-viability would mean a moral breaking point (table 3, quotes 62–63), others felt that even non-viable entities may warrant a certain degree of protection (table 3, quote 61). Lay participants seemed less preoccupied with philosophical questions about moral status and more concerned about viable hELS effectively being used for reproductive purposes, as mentioned before (table 3, quotes 27, 29–30, 59–60).

On both accounts, the question of how to deal with the present-day uncertainty regarding the developmental potential of hELS became paramount. Here, participants favoured a precautionary approach (table 3, quotes 64–65), but what this would require in terms of regulations was less clear. Should policymakers enforce arrested development by legally binding the programming of so-called “suicidal genes” in hELS? Not all participants were convinced, as so doing might only provide a false sense of security and ultimately effectively create a human being with a shortened lifespan (table 3, quote 64).

#### Regulation Should Enforce Public Involvement

Participants were also notably outspoken about the importance of factoring public involvement into the regulation of hELS research. When asked what they considered the most important issues and considerations to be taken on board, lay participants expressed a wish to be more informed about hELS, as well as more involved in the course of the field’s (legislative) future (table 3, quotes 66–69). The view that legislation “must, of course, also be a kind of reflection of what society thinks” (table 3, quote 68), was also shared by professionals. They believed that public engagement is key for both embryo and hELS research to be societally acceptable, not only because lack of it threatens to undermine one’s trust in science and democratic control but also because there is no other context available for addressing the ethical issues at stake (table 3, quotes 70–71).

## Discussion

To our knowledge, this qualitative study is the first to explore lay and professional perspectives on research with hELS empirically. In what follows, we discuss our findings therefore in relation to broader fields of related research (i.e., human embryo research, stem cell research, and synthetic biology), referring where possible to more directly related fields as well (i.e., organoid technology). We conclude by reviewing the limitations of the study, the avenues for further research it opens, and some of its possible implications for the Dutch policy context.

### Common Ground

The first theme underscored the spectrum of perspectives commonly found in related fields of research, with public views often ranging between positive, ambivalent, and negative perspectives (Pauwels 2009, 2013; Ancillotti, et al. 2016; Avellaneda and Hagen 2016; Gouman, Vogelezang, and Verhoef 2020). The fact that these differences were also prevalent in the focus group with professionals suggests that they are not the product of a knowledge deficit concerning science, supporting the view that scientific knowledge does not necessarily cultivate a more positive attitude toward particular avenues of research (Priest, Bonfadelli, and Rusanen 2003; Akin, et al. 2017). At the same time, this group was notably less prone to cynicism than lay groups were. While professionals worried about the occasional “cowboy” (e.g., Jiankui He) but had consensual confidence in the governance mechanisms already embedded in science, lay participants associated hELS research more often with severe dystopias (e.g., The Boys from Brazil (table 3, quote 27)) and showed greater concern about hostile intentions on both individual (i.e., “mad-scientists,” e.g., Josef Mengele (table 3, quote 15)) and institutional levels (e.g., foreign governments (table 3, quote 15)). The qualitative difference between the professionals’ milder and consensual concern for “misuse” and the lay participants’ more severe and discordant concern for “abuse” may relate to what the literature describes as “deference to scientific authority”, i.e., the “stable, long-term reliance on the scientific process and its application” (Akin, et al. 2017, 291). The professionals’ explicit mention that their confidence in hELS research arose from their familiarity with the broader “processes, norms, and structures of the scientific enterprise” (Scheufele 2013, 14044), of which governance mechanisms (e.g., peer-review, ethics committees) are an intrinsic part, seems to support this thesis. Notwithstanding, there may also be other factors influencing these differences. Educational attainment and trust in other institutional bodies, for instance, have previously been found to play a significant role in the Dutch public’s trust in science (van den Broek-Honingh and de Jonge 2018), which could arguably also help explain the qualitative contrast we found between lay and professional participants. From a policy perspective, these findings suggest that it might be wise (for the Dutch government) to inform people not only about the nature of scientific advancements but also, and perhaps more importantly, about the governance mechanisms in which that scientific enterprise is embedded. Without this knowledge, disproportionate concerns about emerging (bio)technologies are more likely.

The second theme showed that most professional and lay participants are receptive to research with hELS as long as the field is regulated. With the exception of a few participants in lay and professional groups, this applied regardless of the (conceptual and moral) qualification of these structures (Pereira Daoud, et al. 2022). Calls for governance are common in synthetic biology and related fields of research, including organoid research (Boerset al. 2018; Haselager, et al. 2020; Lensink, et al. 2021; Bollinger, et al. 2021), and possibly related to the lay participants’ sense of biotechnological ineluctability that we and others (Ancillotti, et al. 2016) have found. Despite the foregoing differences in confidence between lay and professional groups, both groups ended up proposing similar regulatory criteria for having confidence in (the regulation of) hELS research. First, and as previously found by Pauwels (2009), the domain of application played a decisive role in the acceptability of hELS research. Here, two domains were especially contentious: (i) commercial applications, which are known to affect public attitudes towards related emerging biotechnologies (Critchley 2008; Critchley, Bruce, and Farrugia 2013), including organoid-technology in the Netherlands (Boers, et al. 2018; Lensink, et al. 2021), and (ii) reproductive applications, which we discuss in the next section. Second, and in support of the recommendations of Aach and colleagues (Aach et al. 2017), regulating the development of morally concerning features in hELS was deemed crucial by participants, though it remains to be established whether the features they suggested—i.e., developmental potential and (specific features in) organogenesis—should be considered as such (Pereira Daoud, et al. 2022). Finally, and like in previous studies (Ancillotti, et al. 2016; Boers, et al. 2018; Lensink, et al. 2021), collaborative design through public engagement was considered vital in having public confidence in (the regulation of) hELS research. The participants’ call for increased societal engagement involved the three types of motivation previously demarcated by Stirling (2008): “normative—organizing dialogues are good for reasons of democracy, equality or justice; instrumental—building trust, a positive reputation and support; and substantive—moving towards desirable goals, such as environmental quality, public health and human well-being” (Steen and Nauta 2020, 599). The fact that participants in each group voiced these motivations offers proof-of-concept for RRI frameworks in at least two ways. One, it underscores that societal engagement is not only societally desirable but also desired by society; two, it demonstrates that engaging the public is practically feasible, with our results demonstrating that both lay and professional citizens reach similar conclusions.

### Sources of Concern

Taken together, these findings suggest two kinds of concern specifically raised by hELS: (i) general concerns about the technology, and (ii) specific concerns about the application of the technology. Concerns of the general kind arose in relation to what hELS research was perceived to represent, namely, a further step toward potentially deplorable human dominion over life. The shift in moral focus from mere tinkering to creating de novo is commonly found in intersecting fields of research (de Vriend 2006; Pauwels 2009; Ancillotti, et al. 2016), and often contextualized within the extensively discussed “playing God” framework (van den Belt 2009; Dabrock 2009; Douglas and Savulescu 2010; Dragojlovic and Einsiedel 2012; Link 2013; Kaebnick 2014). This framework essentially conveys the view that it would be wrong for humans to (re-)design and create life because so doing would amount to a certain kind of hubris, i.e., a failure to recognize human limitations (Douglas and Savulescu 2010). This view was echoed in principled lay statements about the relationship between humans and the natural world and of the place humans should have within it (table 3, quotes 9–12), which could help explain the “exceptionally strong moral injunctions—strong enough to generate a view that an activity should be flatly banned” (Kaebnick 2014, 146) we found in these groups. The professionals’ emphasis on the two-sidedness of research and importance of deciding what counts as “good” democratically (table 3, quotes 42, 77–78) suggests that their precautionary stance stemmed instead from “the dilemma arising when (…) the risk of harmful use is sufficiently high that it is no longer clear whether that knowledge should be pursued or disseminated” (Douglas and Savulescu 2010, 689).

Specific concerns revolved around the application of the technology in research and reproductive contexts. The research use of hELS was warmly welcomed by participants insofar as hELS do not possess the features that they considered morally concerning. Prominent examples of such features were a heart(beat), central nervous system, and developmental potential (table 3, quotes 61–72). Whereas a central nervous system is widely accepted as a morally important marker because of how it could denote a capacity to experience pain, the moral relevance of a heart or heartbeat remains contested in the ethical literature (Romanis 2019; Colgrove 2020). It is unclear why a heart(beat) would be morally relevant in itself, apart from indicating an ongoing development towards a new human individual. But if that were the reasoning, then it would not be the heart(beat) but rather the potential to grow into a human being that bears moral relevance. Should certain hELS acquire this potential upon further improvement, the question of whether they are to be regarded as human embryos themselves emerges, which would stand in the way of presenting them as morally less-sensitive types of research material. At this point, there is an interesting parallel with the dilemma emerging in the field of human brain organoids. As one commentator has put it, “If it looks like a human brain and acts like a human brain, at what point do we have to treat it like a human brain—or a human being?” (Greely 2020, 35). In light of our findings, further exploration of public perspectives on common ethical issues in brain organoid and hELS research would thus seem warranted (Sawai, et al. 2022). Even though the moral bearing of the embryo’s (or hELS’) “potential” remains a matter of extensive debate (Stier and Schoene-Seifert 2013; Hyun 2013; Piotrowska 2020; Sawai, et al. 2020; Denker 2021), it is noteworthy that the participants’ discussions of the concept (table 3, quotes 66–72) closely aligned with the different positions taken in that scholarly debate. Whereas some participants viewed that potential as granting some but not absolute protection, others argued that its acquisition should be seen as a categorical cut-off point for research with hELS (Pereira Daoud, et al. 2022). These results seem to support precautionary policy trends that distinguish between hELS based on whether or not they can be reasonably expected to lack developmental potential. This distinction is drawn in the Updated Guidelines of the International Society of Stem Cell Research (ISSCR 2021), which recommend subjecting research with hELS that aim to model the “integrated” development of human embryos to greater regulatory oversight and is beginning to be formalized by law in some jurisdictions, including the Netherlands. At the same time, our results also reflect the lack of consensus in the ethical literature about the degree of moral respect that that potential can confer. This means that, even if research with some (“integrated”) hELS were to be regarded as morally equivalent to research with human embryos based on developmental potential, this would by itself still tell us very little about which regulatory conditions and limits we should draw.

The reproductive application of hELS was an unexpected but major point of concern. In the scientific literature, the reproductive application of hELS is usually regarded as too far-off, if not too far-fetched **(**Cyranoski 2019; Nicolas, Etoc, and Brivanlou 2021; Posfai, et al. 2021; Popovic, Azpiroz, and Chuva de Sousa Lopes 2021), and explicitly condemned by the scientific community (ISSCR 2021). This lack of theoretical grounding combined with the already broad scope of our discussions led us to focus exclusively on non-reproductive applications of hELS research. However, despite our conscious effort to guide focus group discussions towards non-reproductive applications, the theoretical possibility of using hELS to create offspring later turned out to be a recurring thread, with participants single-handedly asking about and consensually arguing against the use of hELS in reproductive contexts. The particular reasons to consider the use of hELS for human reproduction as “scary” or “unethical” (table 3, quotes 26, 29) remained largely undetermined, but their explicit associations with cloning (table 3, quotes 27–28, 30) and eugenics (table 3, quotes 58–59) provide telling clues. Whereas references to eugenics seem to indicate a familiar anxiety about societally contentious horizons associated with reproductive selection and enhancement, as thoroughly discussed in the past (Evers 1999; Wilkinson 2010a, 2010b), their combination with concerns related to cloning may be taken to refer to the theoretical scenario of producing genetically modified clones with future hELS technologies (table 3, quote 26). While we cannot establish this hypothesis with certainty, the fact that the reproductive application of hELS is discussed so scarcely in the scientific (and ethical) literature while so vividly present in the minds of lay and professional participants makes it certainly worth investigating further.

## Limitations and Recommendations for Further Research and Debate

This study has several limitations. Due to the timing and explorative aims of the study, priority was given to diversity in views over data saturation. Since these aims focused furthermore only on mapping the range of views on research with hELS rather than the factors influencing these views, the authors did not seek to distinguish between individual characteristics during the data analysis. The use of certain language (“embryo-like structures”) and imagery to denote hELS research during focus group discussions might have contributed to the wide and contrasting range of perspectives identified in the data. The data analysis involved a certain degree of interpretation, meaning that different researchers could have reached different themes and conclusions. Disciplinary bias in the focus group with professionals cannot be ruled out either, and it is worth investigating whether professionals from different disciplines might have different risk perceptions about and attitudes toward hELS research, as this was previously found to play a role (Althaus 2005; Ndoh, Cummings, and Kuzma 2020; de Graeff, Jongsma, and Bredenoord 2021). Since most participants in the professional focus group had previously met each other, interpersonal factors might have been at play without the knowledge of the authors. Similar limitations are conceivable with regard to the pilot focus group, which consisted of participants that were selected from the personal network of the first author. The envisioned scope of the study also evoked noteworthy limitations, specifically in relation to the participants’ concerns about the reproductive application of hELS, which the authors did not aim to explore and were therefore unable to question in more detail. Taken together, these limitations prevent the extrapolation of present findings to larger population groups and limit their utility to purposes of agenda-setting and further research.

Notwithstanding, the importance of involving the public in newly emerging biotechnologies in order to prevent “disproportionate social, ethical and regulatory responses” (Bubela, Hagen, and Einsiedel 2012, 132) is widely acknowledged (Ankeny and Dodds 2008; Zhao, et al. 2015; Zarzeczny and McNutt 2017), including in the Netherlands (NWO 2008). This is especially important in the context of law reform, yet empirical studies on lay and professional perspectives towards hELS research remain understandably lacking due to the field’s recent emergence. The results of this first qualitative exploration of the topic, if interpreted within the context of their limitations, may thus be insightful for researchers and policymakers involved in this field and its regulation.

For researchers in the humanities and social sciences, our results open avenues for both further empirical research and ethical analysis. Apart from how views about the (presently theoretical) reproductive application of hELS would relate to the earlier debate about the ethics of reproductive cloning, this is especially the case with regard to the particular features of moral concern in (different types of) hELS, also taking account of similar debates in the field of human (brain) organoid research (Sawai, et al. 2022). Since this paper did not aim to assess the (moral and logical) validity of these features, further ethics parallel research remains paramount “to separate arguments and values, to recognize whether, and if so which, fallacies have been made, to recognize equivocations and to identify whether there are important questions or positions missing or underrepresented” (Jongsma and Bredenoord 2020, ¶5).

For policymakers, our findings support the need for regulating the emerging field of hELS research. The use of hELS in (important avenues of) research is sometimes regarded as providing a morally neutral alternative to human embryo research, promising scientific progress and its ensuing societal benefits, while avoiding the restrictions and burdens of human embryo research (Pereira Daoud, Dondorp, and de Wert 2021). However, our findings suggest that this is not how our lay and professional participants perceived it. While most participants considered hELS research as potentially beneficial, they unanimously regarded its development as morally charged and therefore in need of regulation. In the Netherlands and other jurisdictions, this process of law reform will require not only reconsidering specific regulations (such as the need to forbid transferring hELS to a womb or to lift the present ban on research embryos, which could preclude some types of hELS research altogether) but also normatively fundamental questions of what embryo legislation aims to protect and why. Involving the public in this process is paramount in developing democratically sound legislation and, to our participants, in having (greater) confidence in the future development of the field and its regulation.

## Supplementary Information

Below is the link to the electronic supplementary material.

Supplementary file1 (DOCX 64 KB)

## Data Availability

The invitational letter, interview guide and pseudonymized transcripts of the focus group discussions with lay citizens are stored in DataVerseNL, “Synthetic embryos: an ethical reflection,” 10.34894/UFM8MN.

## Code Availability

The list of codes generated by lay transcripts in *Atlas.ti* is stored in DataVerseNL, “Synthetic embryos: an ethical reflection,” 10.34894/UFM8MN.
