# Supplementary material for: The Ethics of Stem Cell-Based Embryo-Like Structures: A Focus Group Study on the Perspectives of Dutch Professionals and Lay Citizens
Source: J Bioeth Inq. 2024 Mar 13;21(3):513–42. doi: 10.1007/s11673-023-10325-9 (PMC11652579; doi:10.1007/s11673-023-10325-9)
Supplement: Supplementary file 1 — Supplementary file1 (DOCX 64 KB) [file 11673_2023_10325_MOESM1_ESM.docx]

# **Introduction (5 min)**

## Welcome and Aim of the Meeting:

|  | **(SLIDE #1/8)** Welcome and thanks again for joining us. My name is [BLINDED NAME] and there is my colleague, [BLINDED NAME]. We are today on behalf of [BLINDED UNIVERSITY], where we conduct research into the ethical ramifications of synthetic embryology. **(SLIDE #2/8)** Synthetic embryology is a new field of research that uses stem cells to create increasingly better models of human embryos. These models, so-called ‘embryo-like structures’ or ‘synthetic embryos’, can offer promising medical insights and applications. For example, they can be used to (1) investigate the causes of implantation problems or early pregnancy loss (infertility), which is not possible with human embryos, (2) test the (side) effects of medical drugs, and (3) significantly reduce the need for animal testing. On the other hand, they also raise significant ethical and regulatory questions. We are going to discuss those questions with you today, with the aim of contributing to the development of ethical and legal frameworks for the creation and use of embryo-like structures. We are interested in your opinion, motivation and ideas. This means there are no right or wrong answers and that you do not have to agree with one another. We only ask that you speak freely and give others the space to do so as well. Today’s conversation will be recorded for future reporting and will be treated in the strictest confidence. We also ask that you treat the information discussed here in the strictest confidence. We are happy to send you the publication to which these meetings lead. If you would like to receive it, please be reminded to leave your contact details at the end of the meeting. Finally, we would like to remind you once more that you can withdraw your participation from our study at any time and without providing any reason. Please reach out to us if you decide to do so. |
| --- | --- |

## Discussion Structure and Rules:

|  | **(SLIDE #3/8)** The meeting consists of three parts. Each part begins with a short presentation where we tell you more about how embryos and embryo-like structures relate.  **(SLIDE #4/8)** In order to make sure the discussion goes smoothly and make the best of our limited time, I would like to introduce a few rules during the meeting. Would you please:   - Write your name on the nameplate in front of you; - Preferably, turn off your phone or, if that is not possible, put it on silent;   If there are no further questions, we would like to begin with your initial associations with, or thoughts about, synthetic embryology. I would therefore like to ask you: |
| --- | --- |

# **Discussion (110 min)**

## Introductory Questions (5 min):

**I1. (5 min)** When you think of the possibility to create ‘synthetic embryos’/’embryo-like structures’, what comes to mind? Do you think it is a positive or negative development? *[MOD: Write your name and answers (in keywords) on a piece of paper, read them aloud and hand them in to the moderator]*

**I2. (5min)** Which of the two terms (synthetic embryos or embryo-like structures) do you think best fit the models we are discussing today? Why?

## Conceptual Questions (20 min):

|  | **(SLIDE #5/8)** Generally, the word ‘embryo’ refers to the first eight weeks of development in human embryogenesis. Still, the ‘embryo’ is defined in different ways across international legislations. In some countries, the embryo is defined based on its origin: fertilization (Spain). In other countries, the embryo is defined based on its capacity for further development. This can be either the capacity to *initiate* embryonic development (Australia, “the ability to initiate embryogenesis”) or to *complete* it (the Netherlands, “the ability to grow into a human being”). Unlike embryos, embryo-like structures are created from stem cells. This means they do not require egg or sperm cells. Whether these structures possess the ability to *complete* embryonic development remains to be seen, but they do seem to have the ability to *initiate* it. |
| --- | --- |

**C1. (10 min)** How would you define the human embryo? You may use the aforementioned definitions as well as come up with a definition of your own. If that is too difficult, try to name keywords/criteria that must be met in order to speak of an embryo.

**C2. (10 min)** Are embryo-like structures ‘embryos’? Why (not)? In your opinion, which of the aforementioned criteria/keywords for speaking of an embryo do embryo-like structures do or not meet?

## Normative Questions (50 min):

|  | **(SLIDE #6/8)** The zygote consists of 46 chromosomes: 23 from the egg cell and 23 from the sperm cell. The DNA of an embryo is therefore a unique genetic mix of the DNA of both parents. As you have read in the background information letter, the embryo then develops in a fixed sequence. Unlike embryos, embryo-like structures are genetic copies (i.e., clones) of the stem cells from which they arise. Here we can distinguish between two types:   1. **Embryo-like structures created from embryonic stem cells:** in this case, embryo-like structures would have the DNA of the original embryo from which these stem cells were extracted. 2. **Embryo-like structures created from adult body cells:** in this case, embryo-like structures would have the DNA of the donor of the adult body cells.   In addition, scientists can engineer embryo-like structures to behave or develop differently, for example, by skipping certain traits or having them take place in a different order. |
| --- | --- |

*Text moderator: In front of you lie ten images in random order (a human foetus, a tree, a human zygote, a fish, an 8-week-old human embryo, a chimpanzee, a toddler, an adult person, a mouse and a ‘gastruloid’). You may number these images from 1 to 10 (1 = least worthy of protection, 10 = most worthy of protection) or give multiple images the same number. Write down your chosen order on a piece of paper.*

**N1. (15 min)** Could you read your chosen order aloud and explain your reasoning?

*Text moderator: Imagine working for a hospital as a member of the local ethics committee. Your committee must determine whether and how certain research proposals should be carried out within the hospital.*

*A well-known scientist, Dr. Wilsma, is setting up a nationwide trial in which surplus human embryos (that is, embryos left over from fertility treatments) will be used for research into improving IVF. The hospital you work for asked your committee for permission to participate in this national trial.*

*The use of surplus human embryos for research purposes is permitted by law. The research can provide important scientific knowledge and is methodologically sound.*

**N2. (15 min)** How would you respond to this request? If you need further information in order to make a decision, what information would you need? **To the remnant participants:** What did you like about this response? What would you like to add to it?

*Text moderator: Suppose we change the story slightly.*

*For the research of Dr. Wilsma, it is necessary to be able to study the first stages of embryogenesis. She points out that this is not possible with surplus embryos because by the time they can be used as research material they have past these stages.*

*She asks your hospital to participate in research in which human embryos are specially created to serve as research material. Until recently, this was prohibited by law but the new government dropped the ban on creating research embryos in 2022.*

*The research can provide important scientific knowledge and is methodologically sound.*

**N3. (10 min)** Does this information change your previous response to Dr. Wilsma’s request? Which considerations play a role in your response now?

**N4. (10 min)** What if—instead of human embryos—Dr. Wilsma had asked for your permission to create human embryo-like structures for research purposes? How would you have responded to that request? Which considerations/features play a role in your response?

## Legal Questions (30 min):

|  | **(SLIDE #7/8)** Let us now talk about legislation in the context of embryo research. The permissibility of and conditions for embryo research differ considerably from country to country. In the Netherlands, the frameworks for embryo research are laid down in the Dutch Embryos Act. The Embryo Act allows embryo research under strict conditions. These conditions state that  (1) embryo research is only permitted if it can lead to new insights that are important to public health, which cannot be obtained in any other way,  (2) embryos may not be specially created for research purposes (i.e., only ‘surplus embryos’, left over from fertility treatments, may be used in research),  (3) embryos may not be kept in culture for longer than 14 days (14-day rule), and  (4) embryo research is only permitted after acquiring the approval of the national Medical Ethics Review Committee.  Whether and how these limitations can be applied to embryo-like structures is yet unclear. |
| --- | --- |

**L1. (15 min)** In your opinion, is it acceptable that the law allows the use of surplus human embryos in research? What makes it (un)acceptable? **If acceptable:**

**L1.1.** What do you think about the scope of the research purposes for which surplus embryos may be used (condition 1)?

**L1.2.** To what extent does it matter to you whether the embryos used in research were specially created for that purpose (condition 2)?

**L1.3.** How much time should (surplus) embryos be allowed to be used in research, in your opinion? Should the time limit stay at 14 days or would you prefer a different moment (condition 3)?

**L2. (15 min)** In your opinion, should it be legally allowed to create and use embryo-like structures for research purposes? What would make it (un)acceptable? **If acceptable:** Would you then…

**L2.1. …** prefer that research is conducted with embryo-like structures or with human (surplus) embryos? Which considerations play a role in this preference?

**L2.2.** … conditionally or unconditionally allow research with embryo-like structures? How come? **If conditionally:** What conditions would you like to impose on this type of research? You may use the aforementioned conditions/limits as well as devise new ones yourself. What were the reasons that lead you to choose these conditions/limits?

## Final Questions (5 min):

**F1.** Did this meeting lead you to think differently about ‘synthetic embryos’/’embryo-like structures’? *[Moderator takes out the sheets handed in during the introduction and compares them with the answers of the participants now]*

**F2.** Taking into account all of the issues discussed here today, what do you think are main conclusions and considerations?

**F3.** Are there any other comments and remarks?
